# Decreased Expression of Negative Immune Checkpoint VISTA by CD4+ T Cells Facilitates T Helper 1, T Helper 17, and T Follicular Helper Lineage Differentiation in GCA

**DOI:** 10.3389/fimmu.2019.01638

**Published:** 2019-07-16

**Authors:** Rebeca Hid Cadena, Rosanne D. Reitsema, Minke G. Huitema, Yannick van Sleen, Kornelis S. M. van der Geest, Peter Heeringa, Annemieke M. H. Boots, Wayel H. Abdulahad, Elisabeth Brouwer

**Affiliations:** ^1^Department of Pathology and Medical Biology, University of Groningen, University Medical Center Groningen, Groningen, Netherlands; ^2^Department of Rheumatology and Clinical Immunology, University of Groningen, University Medical Center Groningen, Groningen, Netherlands

**Keywords:** immune checkpoints, V-domain immunoglobulin-containing suppressor of T cell activation (VISTA), programmed death-1 (PD-1), vasculitis, giant cell arteritis

## Abstract

Loss of immune checkpoint (IC) Programmed Death-1 (PD-1) and PD-Ligand1 (PD-L1) expression has been implicated in the immunopathology of Giant Cell Arteritis (GCA). The contribution of the negative immune checkpoint V-domain Immunoglobulin-containing suppressor of T cell activation (VISTA) to GCA pathology has not yet been studied. The aim of our study was to investigate if expression of VISTA and other IC molecules by peripheral blood (PB) immune cells is modulated in GCA and at the site of vascular inflammation. In addition, we assessed the effect of VISTA-Ig engagement on *in vitro* CD4+ T helper (Th) lineage differentiation. To this end, frequencies of monocytes expressing CD80/86, PD-L1, PD-L2, and VISTA were determined in blood samples from 30 GCA patients and 18 matched healthy controls by flow cytometry. In parallel, frequencies of CD4+ cells expressing CD28, Cytotoxic T-Lymphocyte-associated antigen-4 (CTLA-4), PD-1, and VISTA were determined. Immunohistochemistry was employed to detect VISTA, PD-1, and PD-L1-expressing cells in temporal artery biopsies (TABs) diagnostic of GCA. Furthermore, the effect of VISTA-Ig on *in vitro* CD4+ Th lineage differentiation in patients and controls was determined. Our study shows that frequencies of CD80/CD86+ and VISTA+ monocytes were decreased in treated GCA patients only. Moreover, proportions of PD-1+ and VISTA+ Th cells were significantly decreased in GCA patients. Clear infiltration of VISTA+, PD1+, and PD-L1+ cells was seen in GCA TABs. Finally, VISTA-Ig engagement failed to suppress Th1, Th17, and Tfh lineage development in GCA. Our results indicate that decreased expression of VISTA may facilitate development of pathogenic Th1 and Th17 cells in GCA.

## Introduction

The immune system regulates the immune response by balancing a complex network of cell surface molecules required to defend the body against threats whilst maintaining tolerance to self-antigens. T cell activation is of paramount importance to face novel antigenic challenges. The activation of T cells requires two signals to reach full activation: the first signal occurs through the T cell receptor (TCR), which recognizes a cognate peptide antigen when bound to the major histocompatibility complex (MHC) molecules on the surface of antigen presenting cells (APCs). The second signal is provided by immune checkpoint (IC) molecules also known as co-stimulatory and co-inhibitory molecules which determine cell activation or anergy. The magnitude of the response will be proportional to the sum of molecular pathways that strengthen or weaken the immune responses (1). Thus, failure of immune checkpoint regulation may lead to the development of T cell-mediated autoimmune diseases such as Giant Cell Arteritis (GCA).

GCA is the most frequent form of primary vasculitis in the elderly population. Both age-related restructuring of the immune system together with the remodeling processes of the vascular wall facilitate the development of the disease (2). Defective immune responses in age-related immune disorders such as GCA are not fully understood, nevertheless, considerable progress has been made over the last decades identifying CD4+ T cells and monocytes/macrophages as pivotal players in the pathogenic process of GCA (3–7). Briefly, the immunopathological model of GCA occurs as follows: in vasculitic lesions, vascular dendritic cells (vasDCs) are easily activated due to reduced expression of PD-L1 (8). Signals mediated via activated vasDCs are responsible for the recruitment of T cells into the vessel wall. Once recruited, T cells are locally activated by the same vasDCs and are polarized toward Th1 and Th17 development by Th1- (IL-12) and Th17- (IL-1b, IL6, IL23) driving cytokine cues. Th1 and Th17 cells produce large amounts of IFN-ɤ and IL-17, respectively, which favor a state of chronic inflammation and the subsequent recruitment of CD8+ T cells and monocytes into the vessel wall. These monocytes differentiate into macrophages amplifying vascular inflammation, damage, and ultimately triggering the vascular remodeling processes (3, 4, 6, 7, 9).

Thus, both the Th1 and Th17 axes are important contributors to disease activity and inflammation in GCA (10–13). In addition, IL-21-producing CD4+ T cells have been reported to correlate with disease activity and to contribute to Th1 and Th17 expansion in GCA (14). The relationship between IC expression modulation and the different Th lineages has been studied both in GCA and in cancer. In cancerous environments, PD-1 blockade augmented Th1 and Th17 responses (15). In a humanized model of vasculitis, PD-1 blockade enhanced vascular inflammation and tissue production of T cell cytokines IFN-ɤ and IL-17 (8). In contrast, little is known about V-domain Immunoglobulin-containing suppressor of T cell activation (VISTA), an inhibitory receptor expressed on myeloid cells and T cells that has been reported to behave as both ligand and receptor to suppress T cell activation (16, 17). Interestingly, Wang et. al. recently proposed that V-Set and Immunoglobulin domain containing 3 (VSIG-3) could also act as a ligand for VISTA delivering a negative signal thereby, inhibiting T cell activation (18). However, information on the role of VISTA in shaping CD4+ T cell differentiation is limited. In healthy donors, VISTA enhanced the conversion of human naïve T cells into FoxP3+ T cells (16), yet, such information is currently lacking in the context of GCA development.

Changes in checkpoint molecule expression have been linked to the development of a chronic pro-inflammatory state facilitating the development of (auto) inflammatory disorders in the elderly (19–21). Particularly in GCA pathogenesis, abnormalities in the PD-1/PD-L1 pathway have been reported (8, 22). Moreover, immune checkpoint inhibitor (ICI) therapy in cancer was found to trigger GCA development (21, 23–25). In an effort to understand the possible added contribution of IC pathways to the dysregulation of CD4+ T cells in GCA, we aimed to 1: investigate the expression of different IC molecules on CD4+ T cells of GCA patients and compare it with age- and sex-matched healthy controls (HCs), 2: assess checkpoint expression at the vascular site in GCA and non-GCA biopsies and 3: determine the effect of VISTA-Ig engagement on CD4+ subset lineage differentiation.

## Materials and Methods

### Study Population

In a cross-sectional study, fresh blood samples were obtained from 30 GCA patients. Fifteen patients were receiving glucocorticoids (GC), i.e., prednisolone. Four of these patients were also receiving methotrexate. The remaining 15 patients were not receiving GC or methotrexate at the time of blood-withdrawal (Table 1). Blood samples were obtained before noon and all donors were non-fasted/took their prednisolone in the morning. GCA diagnosis was either confirmed by a positive temporal artery biopsies (TABs) and/or positive 18F-fluorodeoxyglucose-positron emission tomography-computed tomography (FDG-PET/CT) (Supplementary Table S1A). As controls, we obtained fresh blood samples from 18 sex- and age-matched healthy controls (HCs) who were screened for past or actual morbidities and pharmacological treatments (Supplementary Table S1B).

**Table 1:** Clinical and Laboratory characteristics of GCA patients (Cross-sectional study).

Sex (F/M)	18/12
Age [years, median (range)]	73 (56–85)
ESR [mm/h, median (range)]	26 (4–104)
CRP [mg/l, median (range)]	5.5 (0.3–134)
Active/remission	13/17
Receiving GC/not receiving GC	15/15
Methotrexate	4
Diagnostic method	10/14/6
Biopsy/PET/combination (biopsy + PET)	
Clinical manifestations at time of diagnosis cranial/systemic/combination	9/5/14
Concomitant polymyalgia rheumatica (PMR)	6
Disease duration [months, median (range)]	18 (0–60)
Claudication	15
Visual ischemia	6

At time of diagnosis, patients were examined for both cranial and/or systemic symptoms. For cranial symptoms, patients had to present one of the following: new headache, temporal artery abnormality, scalp tenderness, jaw/tongue claudication, vision loss (ischemic symptom), amaurosis fugax (ischemic symptom), transient ischemic attack (TIA), or cerebrovascular accident (CVA). The following systemic symptoms reflecting GCA disease activity were taken into account: arm/leg claudication or PMR clinic, and/or two of the following symptoms: fever, weight loss, malaise or night sweats. Symptoms were scored only if they could not be explained by other causes (i.e., infection).

Written informed consent was obtained from all study participants. All procedures were in compliance with the declaration of Helsinki. The study was approved by the institutional review board of the UMCG (METc 2012/375 for HC and METc 2010/222 for GCA patients).

### Treatment of the Patients

GCA patients were initially treated with 40–60 mg/day of prednisone. Tapering of prednisone treatment started after 2–4 weeks, based on normalization of clinical signs and symptoms accompanied by normalization of the erythrocyte sedimentation rate (ESR) and/or C-reactive protein (CRP). After 3 months, the median prednisone dose was 15 mg/day. Seventeen GCA patients were in remission after 3 months of GC treatment. Remission was defined based on the absence of clinical signs and symptoms and normal ESR (<30 mm/h) and/or C-reactive protein (CRP) <5 mg/L.

### Staining for Surface Markers (Flow Cytometry)

Fresh blood samples were collected in EDTA anticoagulant tubes and processed within 2 h. The samples were stained in two separate multi-color flow cytometry panels. The first panel (Panel 1) included the following monoclonal antibodies: anti-CD3, anti-CD4, anti-CD45RA, anti-CD25, anti-CD28, anti-CTLA-4, anti-CD279 (PD-1), and anti-VISTA (Supplementary Table S2A). The second multi-color flow cytometry panel (Panel 2) included the following monoclonal antibodies: anti-CD3, anti-CD16, anti-CD14, anti-CD274 (PD-L1), anti-CD273 (PD-L2), anti-CD80, anti-CD86, and anti-VISTA (Supplementary Table S2B). After this, cells were fixed and erythrocytes were lysed using FACS Lysing solution (BD Biosciences, Durham, NC, USA) according to instructions of the manufacturer. Immediately thereafter, samples were measured on a BD LSR-II flow cytometer. Data were collected for at least 1 × 10^5^ cells and analyzed with Kaluza Analysis Software (Beckman Coulter). Proportions of marker positive/negative cell populations were calculated by quadrant dot-plot analysis based on proper isotype controls. In panel 1, CD4+ T cell subsets were identified and gated as previously described (**Figure 4A**) (26). For gating strategy see (Supplementary Figure S1A). In panel 2, classical (CD14^bright^CD16^neg^), intermediate (CD14^bright^CD16+), and non-classical (CD14^dim^CD16+) monocytes subsets were gated as previously described (Supplementary Figure S1B) (27). In parallel, the absolute numbers of CD3, CD4, and CD8 T-cells and monocytes were determined according to the MultiTest TruCount method (BD), as described by the manufacturer. Data were acquired on a FACS Canto-II (BD Biosciences) and analyzed with FACS Canto Clinical Software (BD).

### Effect of VISTA-Ig Engagement on CD4+ T Cell Differentiation

Ninety-six-well flat-bottom plates were coated with purified anti-CD3 (clone OKT3,) at 2.5 μg/mL mixed together with 10 μg/ ml VISTA-Ig (R&D Systems) or control-Ig protein (110-HG, R&D Systems) in PBS for 2 h at 37°C and stored at 4°C overnight. Wells were washed twice with RPMI 1640 before adding cells. Peripheral blood mononuclear cells (PBMC) were isolated from heparinized blood with Lymphoprep (Axis-Shield). Immediately after, CD4+ T cells were isolated by negative selection using the MagniSortTM Human CD4+ T cell Enrichment Kit (Thermo Fisher Scientific, Waltham, MA., USA) according to instructions of the manufacturer. CD4+T cells were plated at 1 × 10^5^ cells per well in RPMI1640 with 10% heat-inactivated fetal bovine serum and gentamycin. Next, soluble anti-CD28 (clone L293, BD Biosciences) at 250 ng/mL was added to the culture media. After 5 days in culture at 37°C, cells were stained with zombie NIR fixable viability kit (Biolegend) according to instructions of the manufacturer and surface marker CD4. Next, cells were fixed and permeabilized with FoxP3 staining buffer set (eBiosciences) followed by intracellular staining for transcription factors: FoxP3, T-bet, RORgt, BCL6, GATA3 (Supplementary Table S2C).

### Immunohistochemistry (IHC)

Temporal artery biopsies (TABs) were obtained from 5 GCA patients before start of GC treatment. The tissue was fixed in formalin and paraffin embedded. Sections were deparaffinized in xylene and rehydrated in graded ethanol. After antigen retrieval in 1 mM EDTA (pH 8) and endogenous peroxidase blocking, sections were incubated with a rabbit monoclonal anti-VISTA antibody (Clone: D1L2G; Dilution: 1:200; Cell Signaling, Danvers, USA) or an isotype control antibody. The primary antibody was visualized using an EnVision+ Kit (HRP Rabbit AEC+, Dako K4009) and sections were counterstained with hematoxylin. Stainings for PD-1 (Clone: MRQ-22; Dilution: ready to use (R.T.U); Ventana) and PD-L1 (Clone: SP263; Dilution: R.T.U; Ventana) were performed in a Benchmark Ultra automated immunostainer (Ventana, Tucson, Arizona) using pre-diluted antibodies and following the manufacturer's protocols (Supplementary Table S2D). Non-GCA TABs (*n* = 5) and human tonsil tissue were used as controls (Temporal arteries were considered negative for GCA if there were no typical histological findings). Stained sections were scanned using a Nanozoomer Digital Pathology Scanner (NDP Scan U10074–01, Hamamatsu Photonics K.K., Hamamatsu, Japan).

Detection of VISTA, PD-1 and PD-L1-expressing cells (irrespective of intensity) in affected temporal artery biopsy areas was semi-quantitatively scored on a five-point scale (0–4): 0 = no positive cells, 1 = occasional positive cells (0–1% estimated positive), 2 = low numbers of positive cells (>1–20%), 3 = moderate numbers of positive cells (>20–50%), 4 = high numbers of positive cells (more than 50%). Affected regions containing infiltrating cells were scored. Scoring was performed by two independent investigators, trained by a pathologist and average scores were calculated.

Double staining was performed in limited number of samples to confirm the co-localization of VISTA with T cells using CD3 or with macrophages using pu.1 (29). MultiVision Polymer Detection system (MultiVision anti-rabbit/AP + anti-mouse/HRP polymers; Thermo Fisher Scientific, Fremont, USA) was used according to the manufacturer's instructions. Binding of primary antibodies was visualized with the chromogens of the MultiVision kit, LVBlue, and LVRed.

### Statistical Analysis

Non-parametric tests were used for data analysis. The Mann-Whitney *U*-test was used to compare data of patients with HC. Paired samples were analyzed with the Wilcoxon signed rank test. Analyses were performed with GraphPad Prism 7.0 software. *P*-values of <0.05 (2-tailed) were considered statistically significant.

## Results

### Reduced Frequencies of Negative IC Molecule Expressing Monocytes in Peripheral Blood of GCA Patients Is Treatment-Related

**Figure 1:**
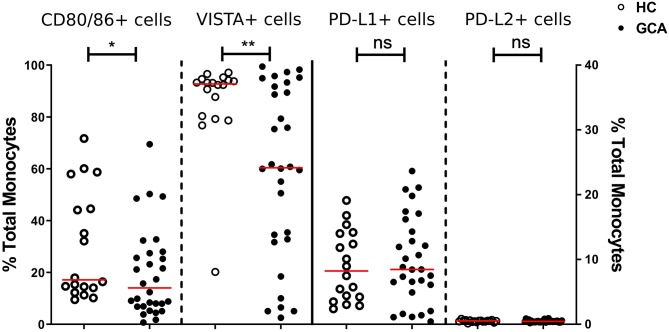
Frequencies of immune checkpoint molecule expressing circulating monocytes of GCA patients and HCs. Frequencies of peripheral monocytes expressing CD80/86 and VISTA (plotted on left y-axis) were decreased in GCA patients (*n* = 30) compared with HCs (*n* = 18). Frequencies of peripheral monocytes expressing PD-1 ligands (PD-L1 and PD-L2) (plotted on right y-axis) were comparable between GCA patients and HCs. The red horizontal lines represent the median. Significant differences by the Mann-Whitney *U*-test are indicated: ^*^P < 0.05, ^**^P < 0.01, ns, non-significant.

**Figure 2:**
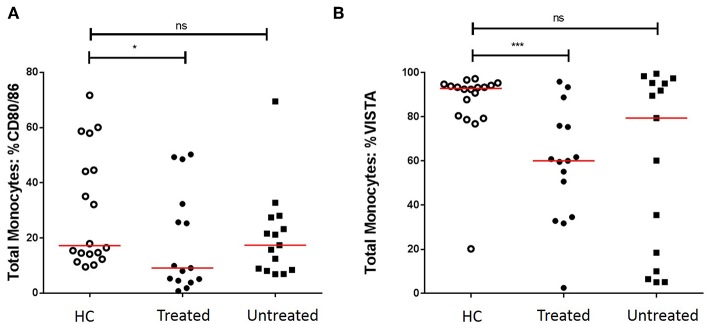
Effect of GC treatment on CD80/CD86 and VISTA-expressing monocytes. **(A)** Decreased proportions of CD80/CD86+ circulating monocytes of treated GCA patients, no differences between untreated GCA patients and HC. **(B)** Decreased proportions of VISTA+ circulating monocytes of treated GCA patients, no differences between untreated GCA patients and HC (HC: *n* = 18, GCA treated: *n* = 15, and GCA untreated: *n* = 15). The red horizontal lines represent the median. Significant differences by the Mann-Whitney *U*-test are indicated: ^*^P < 0.05, ^***^P < 0.001, ns, non-significant.

We first assessed the numbers of monocytes in peripheral blood (PB) of GCA patients and HCs. Total monocyte counts were comparable between groups. Next, we analyzed the expression of CD80/86, as ligands of CD28 and Cytotoxic T-Lymphocyte-associated antigen-4 (CTLA-4) and the expression of PD-L1/PD-L2 as ligands for PD-1 on the surface of monocytes. As VISTA has been reported to behave both as ligand and receptor to suppress T cell activation (30, 31), we measured VISTA+ cells in both panels (panel 1 and 2, Supplementary Tables S2A,S2B). The frequencies of PD-L1 and PD-L2-expressing cells were not different between GCA patients and HCs. Interestingly, the proportions of circulating monocytes expressing CD80/CD86 and VISTA were decreased in GCA patients (Figure 1). As GC treatment has a major effect on immune cells, especially monocytes (27, 32), we analyzed expression in GCA patients who were not receiving GC (*n* = 15) and GCA patients receiving GC (*n* = 15) separately. Indeed, the proportional decrease of monocytes expressing CD80/CD86 and VISTA was treatment related (Figure 2).

### Reduced Frequencies of Negative IC Molecule Expressing CD4+ T Cells in Peripheral Blood of GCA Patients

**Figure 3:**
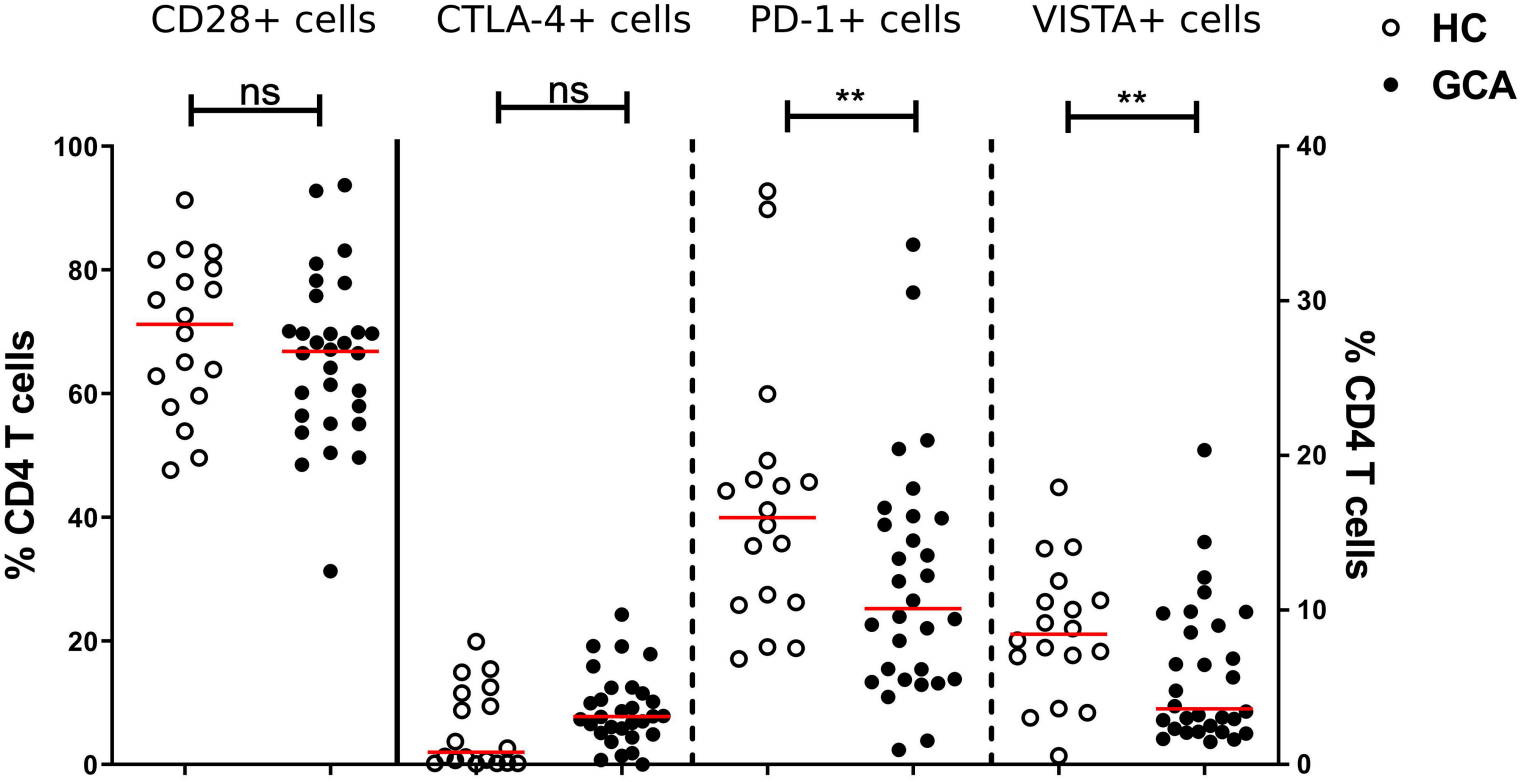
Frequencies of immune checkpoint molecule expressing circulating CD4+ Th cells in GCA patients and HCs. Frequencies of CD28+ (plotted on left y-axis) and CTLA-4+ (plotted on right y-axis) peripheral CD4+ T cells were comparable between GCA patients (*n* = 30) and HCs (*n* = 18). Proportions of PD-1+ and VISTA+ (plotted on right y-axis) peripheral CD4+ T cells were decreased in patients compared with HCs. The red horizontal lines represent the median. Significant differences by the Mann-Whitney *U*-test are indicated: ^**^P < 0.01, ns, non-significant.

Similarly, we measured the absolute numbers and frequencies of CD4+ T cells. No significant differences between GCA patients and HCs were observed (Supplementary Figure S2). Next, we analyzed the frequencies of expression of IC expressing-CD4+ T cells. Proportions of co-stimulatory molecule CD28+ and its outcompeting co-inhibitory molecule CTLA-4+ cells were similar between patients and HCs. We sought to confirm that frequencies of circulating PD-1+ Th cells were reduced in GCA patients (8). Indeed, proportions of these cells in GCA patients were lower than in HCs. In addition, proportions of VISTA+ Th cells were also decreased in GCA patients (Figure 3). Contrary to the outcome seen on IC expression on monocytes, no effect of GC treatment on IC expression by CD4+ T cells was detected. Decreased expression of the negative IC was seen in both patients receiving GCs and not receiving GCs (Supplementary Figure 3).

### Immune Checkpoint Expression in CD45RA and CD25 Defined CD4+ T Cell Subsets

**Figure 4:**
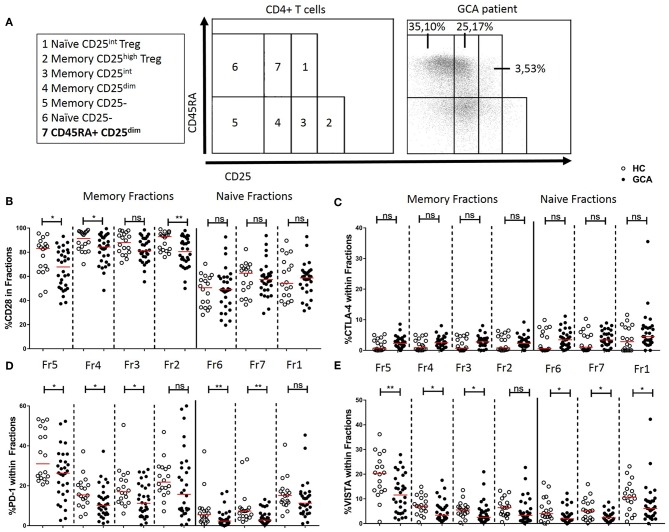
Immune checkpoint expression in CD45RA and CD25 defined CD4+ T cell subsets. **(A)** Flow cytometric gating strategy for analysis of CD45RA and CD25 defined subsets in peripheral blood, as reported by Miyara et al. (28) and adapted by van der Geest (26). Naïve and memory CD4+ T cells subsetted into seven cell fractions. Memory fractions: Memory CD25- (fraction 5), Memory CD25^dim^ (fraction 4), Memory CD25^int^ (fraction 3), and Memory CD25^high^ Treg (fraction 2). Naïve fractions: Naïve CD25- (fraction 6), CD45RA+ CD25^dim^ (fraction 7), and Naïve CD25^int^ Treg (fraction 1). A representative flow cytometry plot is shown for a GCA patient. Frequencies of CD28+ cells **(B)**, CTLA-4+ cells **(C)**, PD-1+ cells **(D)**, and VISTA+ cells **(E)** within memory and naïve fractions of GCA patients (*n* = 30) and HCs (*n* = 18). The red horizontal lines represent the median. Significant differences by the Mann-Whitney *U*-test are indicated: ^*^P < 0.05, ^**^P < 0.01, ns, non-significant.

Next, we determined the frequencies of IC molecule-expressing cells within functionally distinct populations of naïve and memory CD4+ Th cells by CD45RA and CD25 expression (Figure 4A) according to Miyara et al. (28) and adapted by van der Geest et al. to add one age-associated subset (26). The memory fractions include Memory CD25- (fraction 5), Memory CD25^dim^ (fraction 4), Memory CD25^int^ (fraction 3), and Memory CD25^high^ Treg (fraction 2). Within fractions 5 and 4, frequencies of CD28+, PD-1+, and VISTA+ cells were decreased in GCA patients compared to HCs. Memory CD25^int^ (fraction 3) which has intermediate expression of CD25 but is considered as a non-regulatory T cell subset, showed a decrease in the proportions of PD-1+ and VISTA+ cells of GCA patients compared to HCs. Interestingly, within fraction 2, the activated Treg subset, we found unchanged frequencies of cells expressing the negative IC molecules measured, but a decrease in proportions of cells expressing the positive co-stimulator CD28 in GCA patients compared to HCs, thereby suggesting a potentially decreased Treg activity (Figure 4). Within the naïve fractions we identified Naïve CD25- (fraction 6), Naïve CD25^int^ Treg (fraction 1) from the Miyara classification plus the age-associated subset CD45RA+ CD25^dim^ (fraction 7) cells which have been shown to represent a broad and functional reservoir of naïve T cells in the elderly population (26). Within these naïve fractions, frequencies of VISTA+ cells were decreased in GCA patients compared to HCs. Proportions of PD-1–expressing cells were also significantly decreased in conventional naïve T cell fractions 6 and 7 but not in naïve CD25^int^ Treg (fraction 1). Frequencies of CTLA-4-expressing cells within the memory and naïve fractions were comparable between GCA patients and HC (Figure 4C).

### Increased Numbers of VISTA+, PD-1+, and PD-L1+ Cells in Infiltrates of GCA TAB

**Figure 5:**
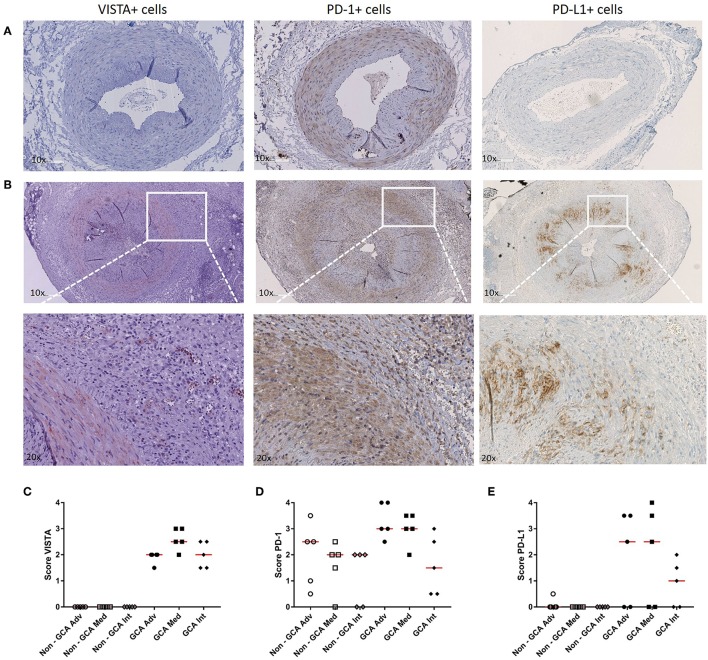
Increase in VISTA+, PD-1+, and PD-L1+ cells in temporal artery biopsies of GCA patients. **(A)** Immunohistochemical staining for VISTA, PD-1, and PD-L1 in non-GCA temporal artery biopsies (TABs) and **(B)** in representative TABs diagnostic of GCA. **(C–E)** Semi-quantitative mean scores of VISTA+, PD-1+, and PD-L1+ cells in inflammatory areas of GCA TABs (*n* = 5) and non-GCA TABs (*n* = 5). Scores are given for the adventitia (Adv), media (Med, infiltrating cells only), and intima (Int). Data are presented as scatter plots. The horizontal red lines represent the median.

As we found PD-1 and VISTA to be modulated in Th cells from GCA patients, we next assessed expression of these checkpoints, including the ligand PD-L1, at lesional sites using immunohistochemistry on GCA diagnostic TABs (Figure 5 and Supplementary Figure S4 for isotype control staining). We found VISTA-expressing cells to be increased within the infiltrates of the adventitia, media and intima layer of the vessel wall when compared to non-GCA biopsies (Figure 5C). PD-1-expressing cells were found mostly within the infiltrates of the medial layer of the vessel wall (Figure 5D). Finally, PD-L1-expressing cells were also increased in GCA biopsies compared with non-GCA TABs (Figure 5E). In addition, double staining for VISTA/CD3 and VISTA/pu.1 confirmed that T cells as well as macrophages in the vascular wall express VISTA (Supplementary Figure S5).

### CD4+ T Cells of GCA Patients Are Unresponsive to VISTA-Ig Engagement

**Figure 6:**
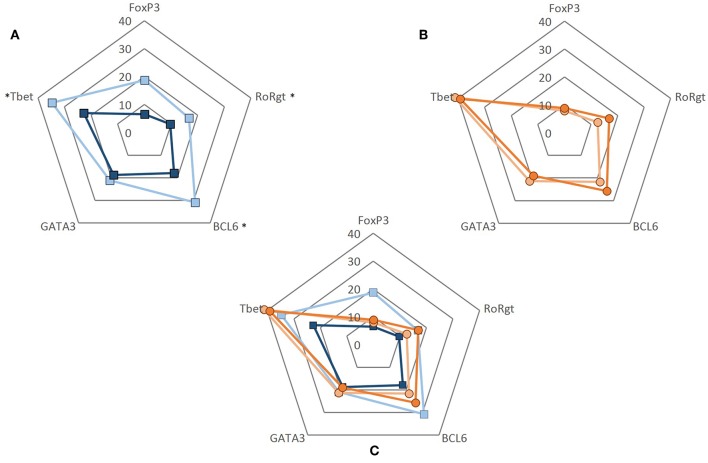
CD4+ T cell differentiation upon VISTA engagement. CD4+ T cells were negatively isolated from PBMCs. Cells were cultured in 96-well flat-bottom plates and stimulated for 5 days at 37°C in anti-CD3 (2.5 ug/mL) coated plates simultaneously coated with either control Ig or VISTA-Ig (both 10 ug/mL). Soluble anti-CD28 was added at 250 ng/mL. After 5 days, CD4+ T cell differentiation was assessed by measuring the expression of intracellular transcription factors (T-bet, RORɤt, BCL6, GATA3, and FoxP3). **(A)** Frequency of transcription factor expression of CD4+ T cells from healthy controls when engaged to control Ig (light blue) and VISTA-Ig (dark blue) (*n* = 6). **(B)** Frequency of transcription factor expression of CD4+ T cells from GCA patients when engaged to control Ig (light orange) and VISTA-Ig (dark orange) (*n* = 6). **(C)** Radar plot of the frequency of transcription factor expression of CD4+ T cell from HC (blue) and GCA patients (orange) combined. Individual values represent the median. Significant differences by Wilcoxon signed-rank test are indicated: ^*^P < 0.05, ns, non-significant.

To evaluate whether the decrease in the frequencies of VISTA+ cells seen in GCA patients would translate into a functional effect, we performed experiments using recombinant human VISTA on human T cells. To this end, VISTA-Ig or control Ig was immobilized on plates with anti-CD3 (OKT3) and soluble anti-CD28 was added to provide appropriate co-stimulatory signaling. Unstimulated samples served as controls. Dead cells were excluded from the analysis and gates were set according to unstimulated samples (Supplementary Figure S6). The ability of CD4+ T cells to differentiate into Th subsets (Th1, Th17, Tfh, Th2, and Treg) was assessed by measuring the expression of intracellular transcription factors (T-bet, RORɤt, BCL6, GATA3, and FoxP3) (Supplementary Figure S7). The effect of VISTA-Ig engagement on CD4+ T cell differentiation in HCs showed a decrease of T-bet, RORɤt and BCL6-expressing cells when compared to Ig control engagement (Supplementary Figures S8, S9) The expression of GATA3 and FoxP3 remained unchanged. On the other hand, applying the same differentiation conditions to CD4+ T cells of GCA patients, we found that VISTA-Ig engagement failed to modulate any of these transcription factors, hence facilitating Th1, Th17, and Tfh lineage differentiation (Figure 6).

## Discussion

We found that expression of IC molecules PD-1 and VISTA is reduced in both memory and naïve CD4+ T cells of GCA patients. Our functional studies show that VISTA plays a role in skewing CD4+ lineage differentiation and that VISTA-Ig engagement failed to suppress Th1, Th17, and Tfh lineage development in GCA. Reduced VISTA expression may thus favor the expansion of pathogenic Th1 and Th17 responses in GCA patients. Our VISTA data strengthen the hypothesis of failing immune regulation in GCA development.

The molecular mechanism by which VISTA exerts its inhibitory effects is not yet fully understood. However, there have been several reports of VISTA's dual behavior, as a ligand and receptor, suppressing T cell activation (33–36). We here provide evidence for a role of VISTA in shaping Th lineage differentiation. Previously, VISTA was shown to enhance the conversion of human naïve T cells into FoxP3+ T cells of healthy individuals (16). In our *in vitro* model we did not detect a role for VISTA-Ig in the enhancement of FoxP3+ T cells. However, we show that in GCA, VISTA engagement failed to suppress Th1, Th17, and Tfh differentiation, consequently, reduced VISTA expression may favor the development of pathogenic Th1 and Th17 responses in GCA. Although the contribution of Th1 and Th17 to GCA is clearly established, the contribution of Tfh cells seems counterintuitive as GCA is not characterized by disease-specific autoantibodies. Yet, the IL-21 producing capacity of Tfh cells could contribute to IL-21-mediated expansion of pathogenic Th1 and Th17 in GCA (14). Clearly, the mechanism by which VISTA mediates these effects and the role of IL-21 producing Tfh cells in GCA remain to be established.

The modulation of IC on monocytes was found to be related to GC treatment. GC represent an important therapeutic strategy to treat various inflammatory and autoimmune diseases such as GCA. GC are immunomodulatory agents which regulate gene expression and signaling pathways by binding to intracellular receptors (37, 38). In addition, some of the effects of GC exposure include decreased DC ability to present antigens and mount an optimal T cell response. This is a consequence of GC preventing the upregulation of MHC class II and costimulatory molecules CD80 and CD86 (37, 38). Hence, it is likely that GC modulated these IC on monocytes of GCA patients in our study.

Here, we report lower frequencies of circulating PD-1 and VISTA-expressing CD4+ T cells in GCA patients. This was independent of treatment, suggesting that the IC modulation was likely due to vasculitis-associated inflammation. Our data confirms the previously reported decrease of circulating PD-1+ CD4+ T cells in GCA patients (8). Here, we extend these findings by showing reduced frequencies of VISTA+ Th cells in GCA patients. Moreover, we found reduced frequencies of CD28, PD-1, and VISTA-expressing cells in most memory T cell subsets. Interestingly, memory Tregs showed a decreased proportion of CD28+ cells which could translate into a diminished regulatory activity. Remarkably, we found that frequencies of PD-1 and VISTA-expressing cells were already reduced in all naïve T cell fractions. The latter finding suggests that naïve T cells in GCA may already be imprinted by systemic inflammatory processes although no direct correlation was found between IC decreased expression on immune cells and inflammatory markers (i.e., CRP, ESR). Decreased immunoinhibitory checkpoint expression is thought to be related to persistence of T cell activation. The combination of low CD28 activated Tregs together with decreased negative IC in both memory and naïve T cells might facilitate the activation of effector/autoreactive T cells in GCA. In our *in vitro* study, favoring the stimulation and differentiation of naïve T cells, we found VISTA-Ig engagement to down modulate T-bet, RORɤt and BCL6 lineage differentiation in CD4+ T cells of HCs. In contrast, CD4+ T cells of GCA patients were found to be non-responsive to VISTA-Ig engagement.

We also investigated IC expression in GCA lesional sites. Our data showed, consistent with increased infiltration of the vessel wall in GCA TABs, higher numbers of VISTA+ cells throughout all three layers of the vessel wall. We found higher numbers of PD-1-expressing cells in granulomatous lesions of GCA patients, particularly in the media layer when compared to non-GCA control tissues. Lastly, within the vessel wall of GCA patients, we found higher numbers of PD-L1+ cells. The increase of negative IC within the vessel wall could indicate a futile attempt to decrease immune activation and prevent further damage.

Persistent activation of T cells, especially Th1 and Th17 cells, is a well-known phenomenon occurring in GCA patients (3, 4, 9, 11). Furthermore, our *in vitro* T cell differentiation studies substantiate the notion of persistent activation as VISTA-Ig mediated suppression did not suppress CD4+ lineage differentiation in GCA patients. This could mean that VISTA expression on the surface of these cells is insufficient to properly transmit the negative signal. This may also be true at the site of inflammation. Despite the increase of VISTA-expressing cells in the infiltrated layers of the vascular wall, vascular inflammation and occlusion appears to be an ongoing process. Future validation studies should be performed in an independent larger cohort to confirm our observations.

Growing evidence indicates that the impaired PD-1/PD-L1 axis plays an important role not only in cancer but also in GCA and a variety of autoimmune diseases (39–41). Here, we demonstrate that the VISTA signaling pathway fails to control Th1, Th17, and Tfh lineage differentiation in GCA. The data add to a further understanding of unchecked pathogenic T cell responses in GCA and may aid the development of new therapeutic strategies.

## Data Availability

The datasets generated for this study are available on request to the corresponding author.

## Ethics Statement

Written informed consent was obtained from all study participants. All procedures were in compliance with the declaration of Helsinki. The study was approved by the institutional review board of the UMCG (METc 2012/375 for HC and METc 2010/222 for GCA patients).

## Author Contributions

RH carried out the data gathering, data analysis, figures/tables preparation, and manuscript writing. WA, PH, AB, and EB supervised the experimental design, data analysis, and manuscript writing. All authors provided substantial, direct and intellectual contribution to the work, and also read and approved the final manuscript.

### Conflict of Interest Statement

The authors declare that the research was conducted in the absence of any commercial or financial relationships that could be construed as a potential conflict of interest.
