# Supplementary material for: Decreased Expression of Negative Immune Checkpoint VISTA by CD4+ T Cells Facilitates T Helper 1, T Helper 17, and T Follicular Helper Lineage Differentiation in GCA
Source: Front Immunol. 2019 Jul 16;10:1638. doi: 10.3389/fimmu.2019.01638 (PMC6646729; doi:10.3389/fimmu.2019.01638)
Supplement: Supplementary file 1 [file Data_Sheet_1.docx]

Supplementary Material

## Supplementary Tables

**Supplemental Table S1A.** Characteristics of GCA patient cohort and overview of sample inclusion per patient.

| Patient ID | Sex | Age | Diagnosis | Cell Count PB | CD4 T cell subsets (%) PB | Monocyte Subsets (%) PB | IC Molecules PB | IHC Study | Receiving GC | Methotrexate |
| --- | --- | --- | --- | --- | --- | --- | --- | --- | --- | --- |
| GCA5 | F | 74 | PET-CT + TAB |  |  |  |  | X |  |  |
| GCA6 | F | 79 | PET-CT + TAB | X | X | X | X | X |  |  |
| GCA7 | M | 71 | PET-CT | X | X | X | X |  |  | X |
| GCA8 | F | 64 | PET-CT + TAB | X | X | X | X | X |  |  |
| GCA10 | F | 77 | PET-CT |  | X | X | X |  |  |  |
| GCA11 | M | 83 | PET-CT | X | X | X | X |  | X |  |
| GCA12 | F | 77 | TAB | X | X | X | X |  |  |  |
| GCA13* | M | 60 | PET-CT | X | X | X | X |  | X | X |
| GCA15 | F | 72 | TAB | X | X | X | X |  |  |  |
| GCA17* | F | 84 | PET-CT | X | X | X | X |  | X |  |
| GCA18 | F | 65 | PET-CT | X | X | X | X |  |  |  |
| GCA20 | F | 81 | TAB | X | X | X | X | X |  |  |
| GCA21 | M | 78 | PET-CT + TAB |  | X | X | X |  |  |  |
| GCA22 | M | 68 | PET-CT + TAB |  | X | X | X |  |  |  |
| GCA23* | F | 78 | PET-CT | X | X | X | X |  | X |  |
| GCA26 | M | 66 | PET-CT + TAB | X | X | X | X |  | X | X |
| GCA27 | M | 70 | PET-CT |  | X | X | X |  | X |  |
| GCA28 | F | 67 | PET-CT | X | X | X | X |  | X |  |
| GCA31 | M | 61 | PET-CT | X | X | X | X |  | X |  |
| GCA32 | F | 85 | TAB | X | X | X | X |  | X |  |
| GCA33* | F | 75 | PET-CT | X | X | X | X |  | X |  |
| GCA34 | F | 71 | TAB | X | X | X | X |  | X | X |
| GCA35 | F | 81 | TAB | X | X | X | X |  | X |  |
| GCA36 | F | 63 | PET-CT |  | X | X | X |  | X |  |
| GCA37* | F | 81 | PET-CT | X | X | X | X |  | X |  |
| GCA38* | F | 56 | PET-CT | X | X | X | X |  | X |  |
| GCA39** | M | 75 | TAB | X | X | X | X |  |  |  |
| GCA40** | F | 74 | TAB | X | X | X | X |  |  |  |
| GCA41** | F | 60 | PET-CT + TAB | X | X | X | X |  |  |  |
| GCA42** | F | 79 | TAB | X | X | X | X |  |  |  |
| GCA43** | M | 70 | TAB | X | X | X | X |  |  |  |
| GCA1515 | M | 70 | PET-CT + TAB |  |  |  |  | X |  |  |

GCA: Giant Cell Arteritis. *GCA patients with concomitant PMR. ** GCA patients newly diagnosed and untreated. PB: peripheral blood; M: Male; F: Female; PET-CT: positron emission tomography-computer tomography; TAB: temporal artery biopsy; GC: glucocorticoids; X: sample was included.

**Supplemental Table S1B.** HC cohort and overview of sample inclusion per donor.

| ID | Sex |  | Age | Cell Count PB | CD4 T cell subsets (%) PB | Monocyte Subsets (%) PB | Immune Checkpoint Molecules PB |
| --- | --- | --- | --- | --- | --- | --- | --- |
| SEN2 | F |  | 71 |  | X | X | X |
| SEN3 | F |  | 77 |  | X | X | X |
| SEN25 | F |  | 78 |  | X | X | X |
| SEN27 | F |  | 67 | X | X | X | X |
| SEN28 | M |  | 71 | X | X | X | X |
| SEN41 | M |  | 85 | X | X | X | X |
| SEN44 | M |  | 83 | X | X | X | X |
| SEN45 | F |  | 86 | X | X | X | X |
| SEN48 | M |  | 71 | X | X | X | X |
| SEN53 | M |  | 81 | X | X | X | X |
| SEN54 | F |  | 82 | X | X | X | X |
| SEN55 | F |  | 72 |  | X | X | X |
| SEN66 | M |  | 77 | X | X | X | X |
| SEN68 | F |  | 76 | X | X | X | X |
| HCGRAIL7 | M |  | 57 | X | X | X | X |
| HCGRAIL15 | F |  | 54 |  | X | X | X |
| HCGRAIL24 | M |  | 64 |  | X | X | X |
| HCGRAIL30 | F |  | 63 | X | X | X | X |

HC: Healthy Control; PB: peripheral blood; M: Male; F: Female; X: sample was included

**Supplemental Table S2A.** Primary antibody information used in flow cytometry (Panel 1).

| **Antibody** | **Conjugated Fluorochrome** | **Clone** | **Supplier** |
| --- | --- | --- | --- |
| anti-CD3 | AF700 | UCHT1 | BD Biosciences, Franklin Lakes, NJ, USA. |
| anti-CD4 | PerCP Cy 5.5 | OKT4 | BioLegend, San Diego, CA, USA. |
| anti-CD25 | BV421 | BC96 | Biolegend |
| anti-CD45RA | BUV737 | HI100 | BD Biosciences |
| anti-CD28 | APC-H7 | CD28.2 | BD Biosciences |
| anti-CD152 (CTLA-4) | PE- Cyanine7 | 14D3 | eBioscience, San Diego, CA, USA. |
| anti-VISTA | PE | 730804 | R&D Systems, Minneapolis, MN, USA. |
| anti-CD279 (PD-1) | BV605 | EH12.2H7 | BioLegend |

**Supplemental Table S2B.** Primary antibody information used in flow cytometry (Panel 2).

| **Antibody** | **Conjugated Fluorochrome** | **Clone** | **Supplier** |
| --- | --- | --- | --- |
| anti-CD3 | AF700 | UCHT1 | BD Biosciences, Franklin Lakes, NJ, USA. |
| anti-CD4 | PerCP Cy 5.5 | OKT4 | BioLegend, San Diego, CA, USA. |
| anti-CD16 | BUV395 | 3G8 | BD Biosciences |
| anti-CD14 | BV605 | M5E2 | Biolegend |
| anti-CD86 | BB515 | 2331 (FUN-1) | BD Biosciences |
| anti-CD80 | BB515 | L307.4 | BD Biosciences |
| anti-CD274 (PD-L1) | PE-Cyanine7 | 29E.2A3 | Biolegend |
| anti-CD273 (PD-L2) | PerCP-Cyanine5.5 | MIH18 | BD Biosciences |

**Supplemental Table S2C.** Primary antibody information used in flow cytometry (Panel 3: Intracellular staining of transcription factors).

| **Antibody** | **Conjugated Fluorochrome** | **Clone** | **Supplier** |
| --- | --- | --- | --- |
| Zombie NIR™ Fixable Viability Kit | Red Laser (633 nm) | NA | BioLegend, San Diego, CA, USA. |
| anti-CD4 | BUV395 | SK3 | BD Biosciences, Franklin Lakes, NJ, USA. |
| anti-FoxP3 | BV421 | 206D | BioLegend |
| anti-RORgt | PE | Q21-559 | BD Biosciences |
| anti-Tbet | PerCP Cy5.5 | 4B10 | Invitrogen, Carlsbad, CA, USA. |
| anti-BCL6 | AF488 | K112-91 | BD Biosciences |
| anti-GATA3 | AF647 | L50-823 | BD Biosciences |

**Supplemental Table S2D.** Primary antibody information used in immunohistochemistry

| **Antibody** | **Isotype** | **Clone** | **Supplier/Cat #** | **Dilution** | **Antigen retrieval** |
| --- | --- | --- | --- | --- | --- |
| anti-VISTA | Rabbit IgG | (D1L2G™)XP | Cell Signaling Technology (CST), Danvers, MA, United States | 1:200 | 1mM EDTA pH=8 |
| anti-CD279 (PD-1) | Mouse IgG | MRQ-22 | Ventana, Tucson, Arizona, USA | Ready to use  (R.T.U) | Cell Conditioning buffer pH 8.4 |
| anti-CD274 (PD-L1) | Rabbit IgG | SP263 | Ventana | R.T.U | Cell Conditioning buffer pH 8.4 |

**Supplementary Figures**

**Supplemental Figure S1A. Flow cytometric gating strategy to measure IC molecules in CD4+ T cells.** Lymphocytes were gated using FCS/SSC, from the entire lymphocyte population, only single cells were gated to identify CD4+ T cells using the CD3/CD4 plot. Within the CD4+ T cells, IC molecules CD28, CTLA-4, PD-1 and VISTA proportions were calculated based on proper isotype controls. Representative flow cytometry plots are shown for a HC and a GCA patient.


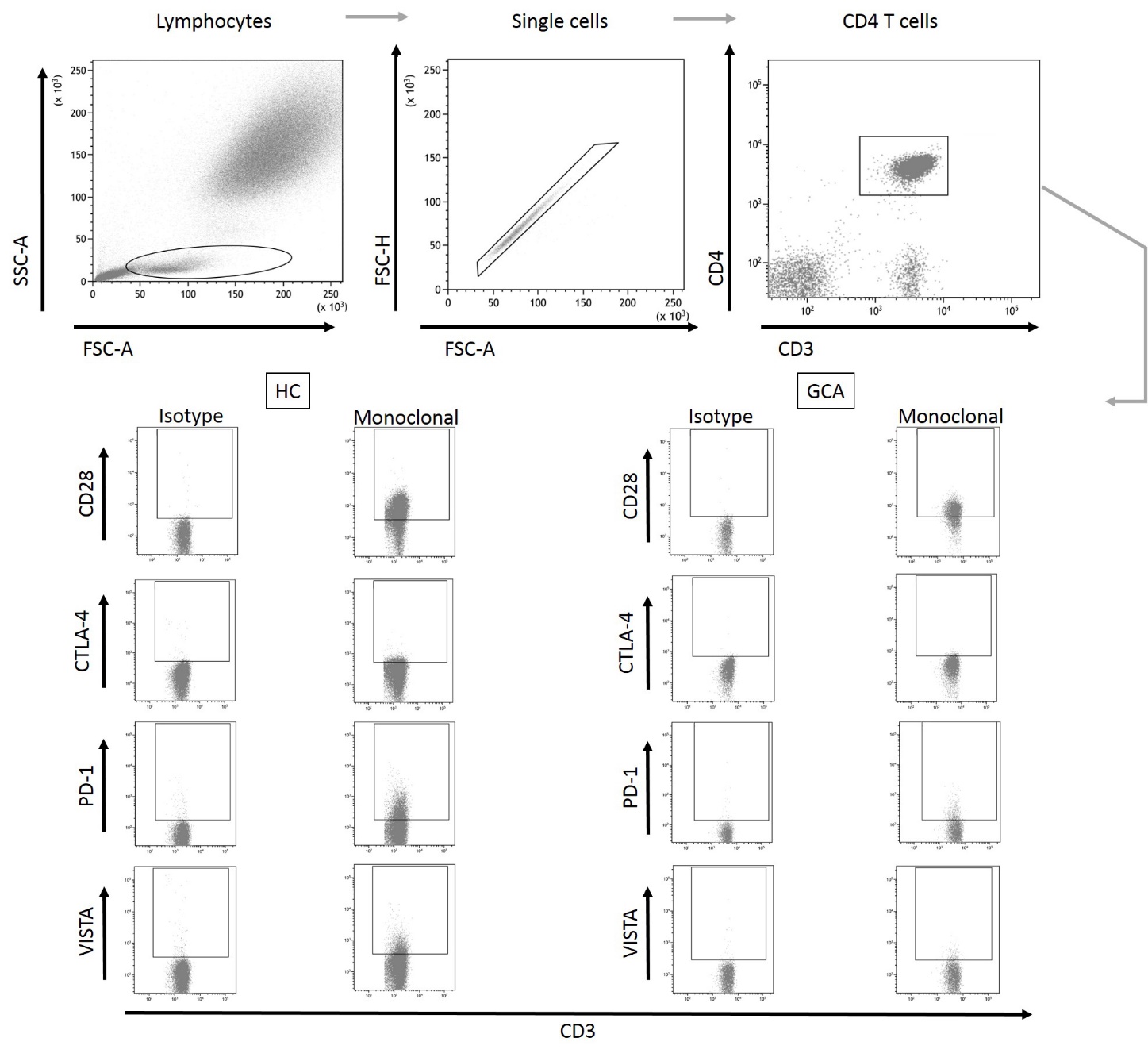


**Supplemental Figure S1B. Flow cytometric gating strategy to identify monocyte subsets.** Monocytes subsets were identified based on CD14 and CD16 expression as classical (CD14^bright^CD16^neg^), intermediate (CD14^bright^CD16+) and non-classical (CD14^dim^CD16+) monocytes. Representative flow cytometry plots (equal number of events) are shown for a HC and a GCA patient.


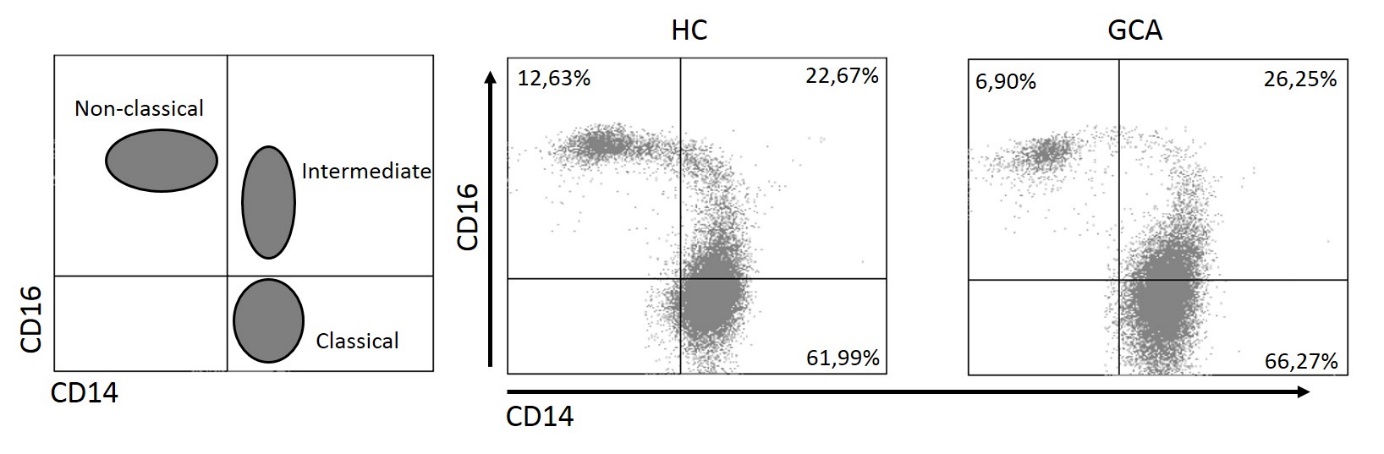


**Supplemental Figure S2: No differences in CD4+ T cells absolute counts or proportions.** (A) Absolute numbers of CD4 T cells of healthy controls (HC, n=12) and GCA patients (n=24). (B) Proportions of circulating CD4 T cells of HCs (n=18) and GCA patients (n=30). The red horizontal lines represent the median. Significant differences by the Mann-Whitney U test are indicated: ns= non-significant.

**
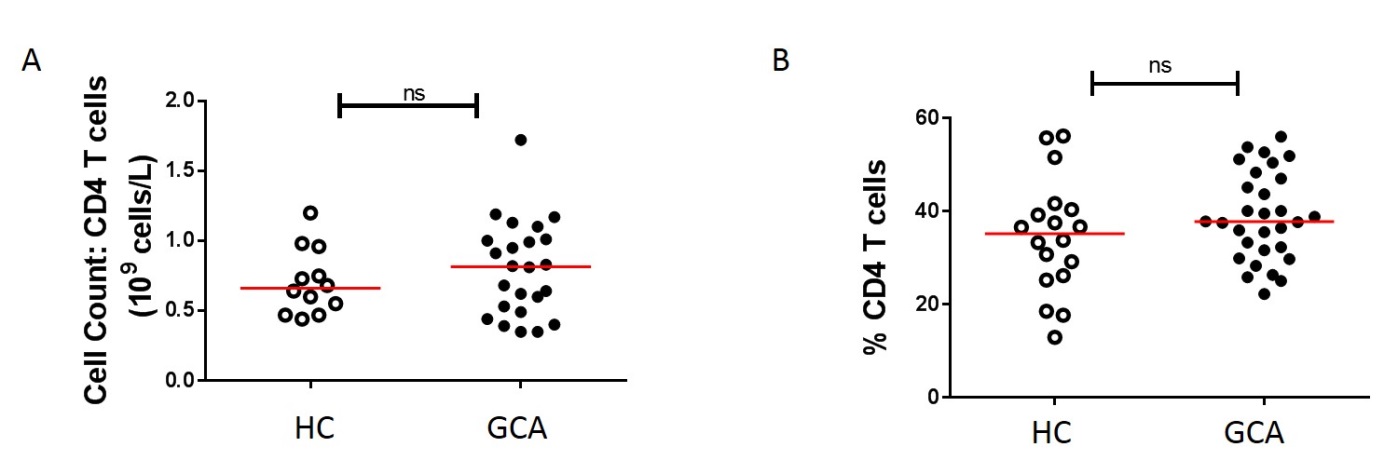
**

**Supplemental Figure S3. No effect of GC treatment on PD-1 and VISTA-expressing CD4+ T cells.** (A) Decreased proportions of PD-1+ circulating CD4+ T cells of treated and untreated GCA patients compared with HC. (B) Decreased proportions of VISTA+ circulating CD4+ T cells of treated and untreated GCA patients compared with HC. (HC: n=18, GCA treated: n=15 and GCA untreated: n=15). The red horizontal lines represent the median. Significant differences by the Mann-Whitney U test are indicated: *P<0.05, **P< 0.01, ns= non-significant.

**
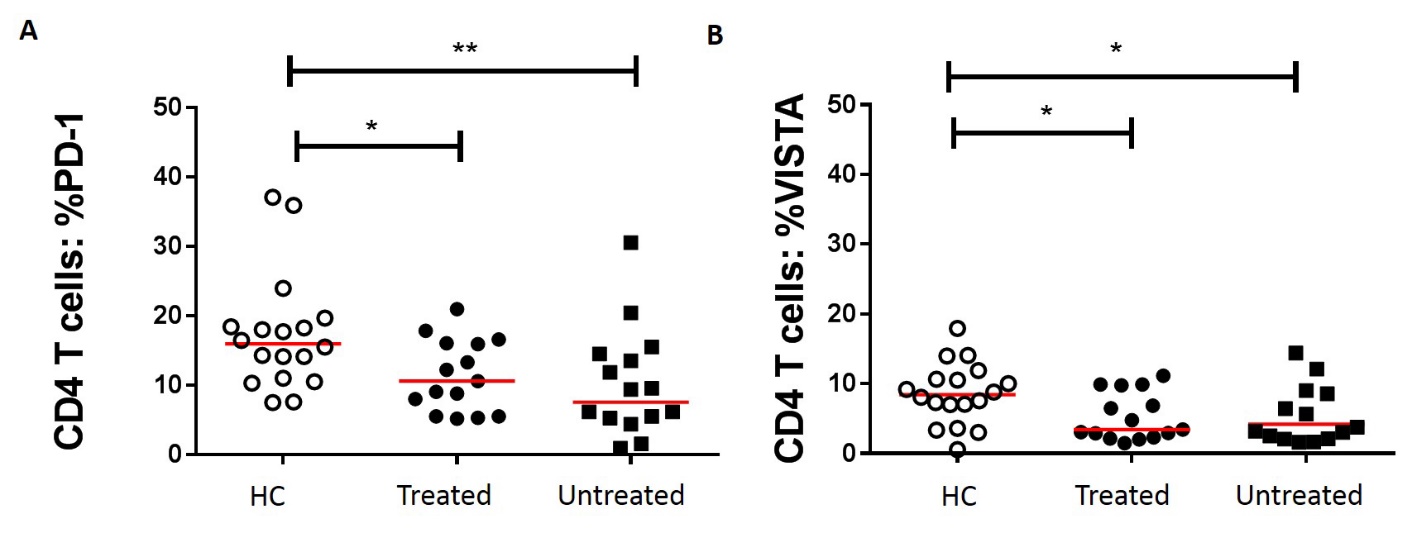
**

**Supplemental Figure S4: Isotype control for immunohistochemistry VISTA staining.** Immunohistochemistry staining for Rabbit IgG in a representative inflamed temporal artery biopsy from a GCA patient.


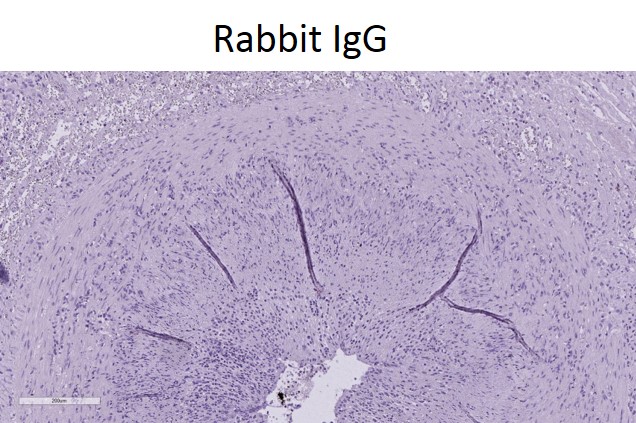


**Supplementary Figure S5: VISTA co-localizes with CD3 and pu.1 in temporal artery biopsies from GCA patients.** (A) Co-localization of VISTA with CD3 and (B) co-localization of VISTA with pu.1 in the temporal artery biopsy of a GCA patient. Blue= VISTA staining; Red= lineage marker CD3 (T cells) or pu.1 (macrophages). Arrows indicate co-localization of VISTA with T cells (A) or macrophages (B).

**
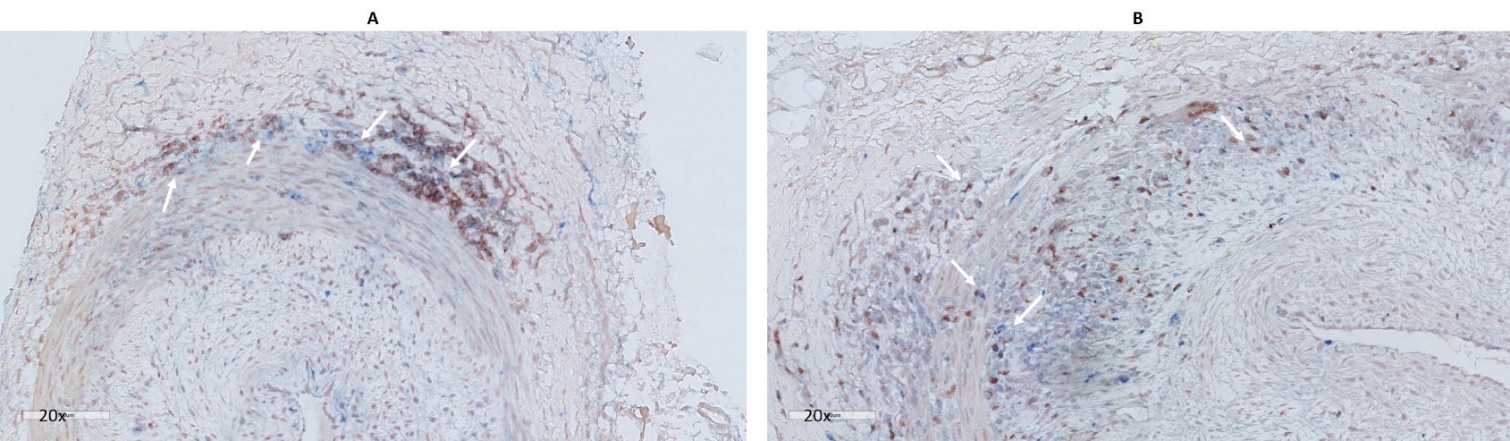
**

**Supplemental Figure S6: Flow cytometric gating strategy of CD4+ T cell differentiation.** Representative flow cytometric plot showing gating of single and alive CD4+ T cells.

**
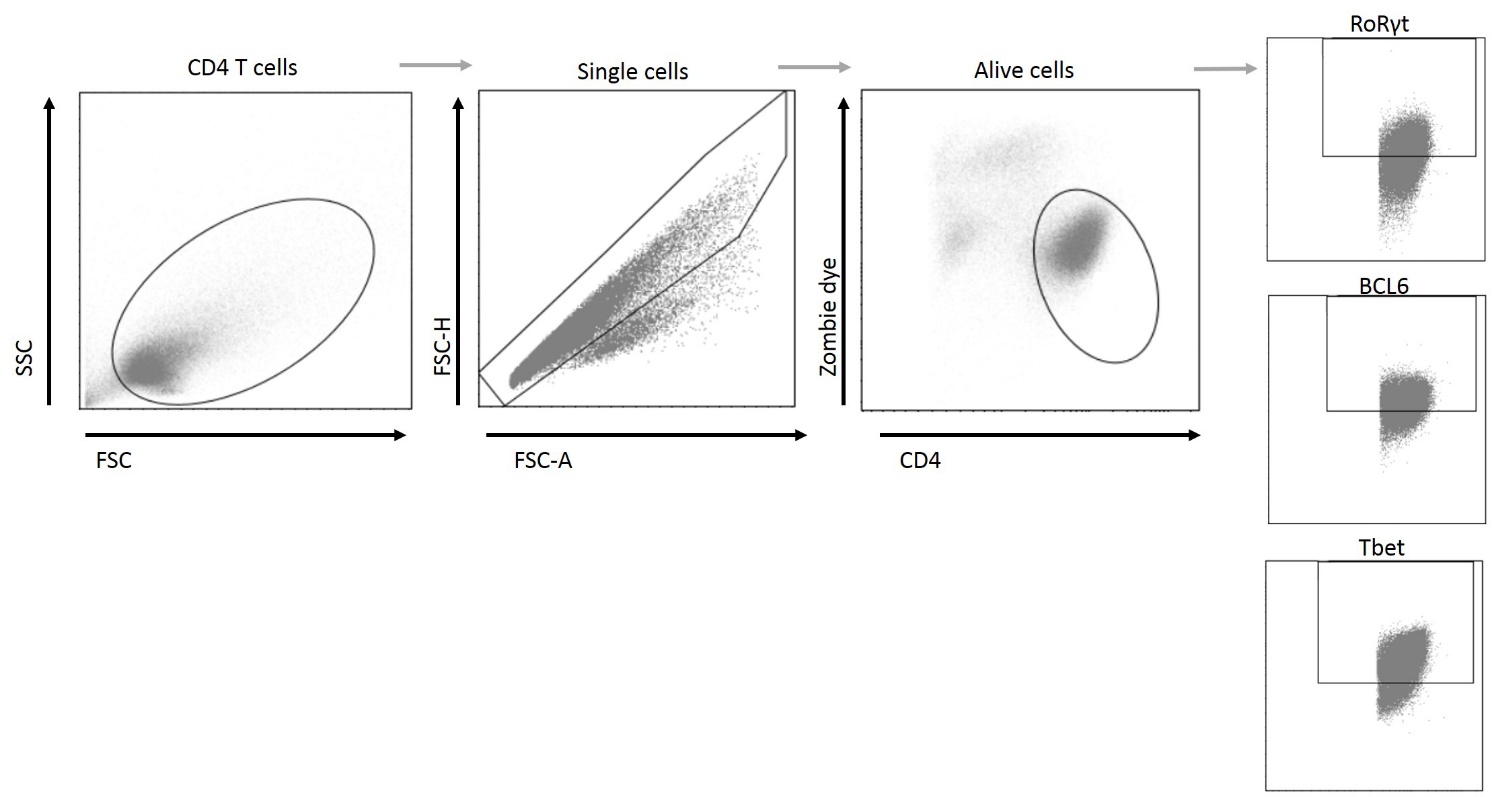
**

**Supplemental Figure S7: Percentages of cells expressing transcription factors within CD4+ T cells of HC and GCA patients.** (A) Percentages of cells expressing FoxP3, RoRɤt, BCL6, GATA3, T-bet within CD4+ T cells of 6 HCs and 6 GCA patients (B) after 5 days of culture with coated anti-CD3 together with control Ig or VISTA-Ig and soluble anti-CD28. Unstimulated samples served as controls. Gates were set according to unstimulated samples and dead cells were excluded from the analysis by using a fixable viability dye. Significant differences by Wilcoxon signed-rank test are indicated: *P<0.05, ns= non-significant.

**
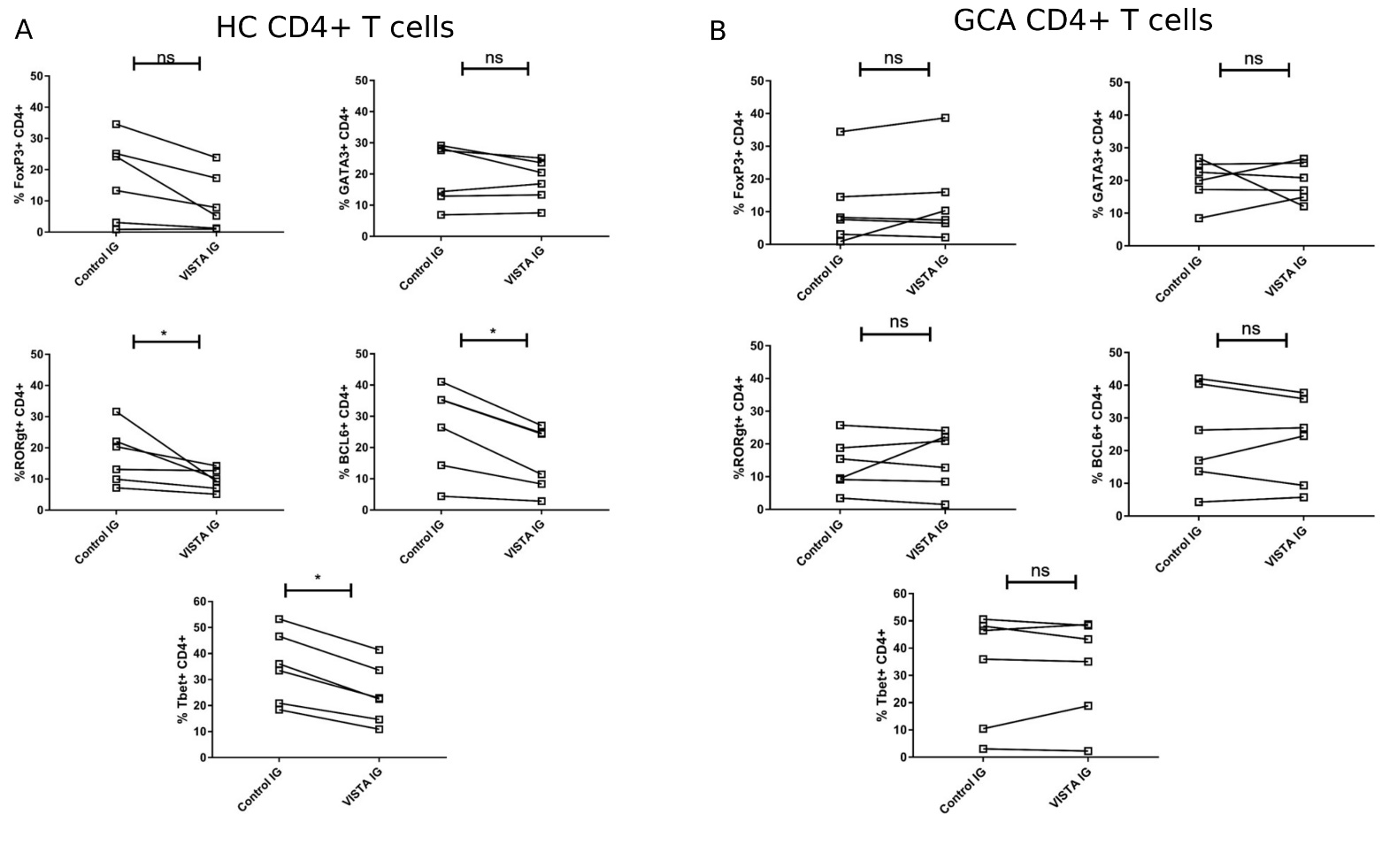
**

**Supplemental Figure S8: CD4+ T cells of GCA patients seem unresponsive to VISTA-Ig engagement.** Representative flow cytometric staining of intracellular RoRɤt, BCL6 & Tbet in CD4+ T cells of a HC and GCA patient after 5 days of culture with coated anti-CD3 together with control Ig or VISTA-Ig and soluble anti-CD28. Unstimulated samples served as controls. Gates were set according to unstimulated samples and dead cells were excluded from the analysis by using a fixable viability dye.

**
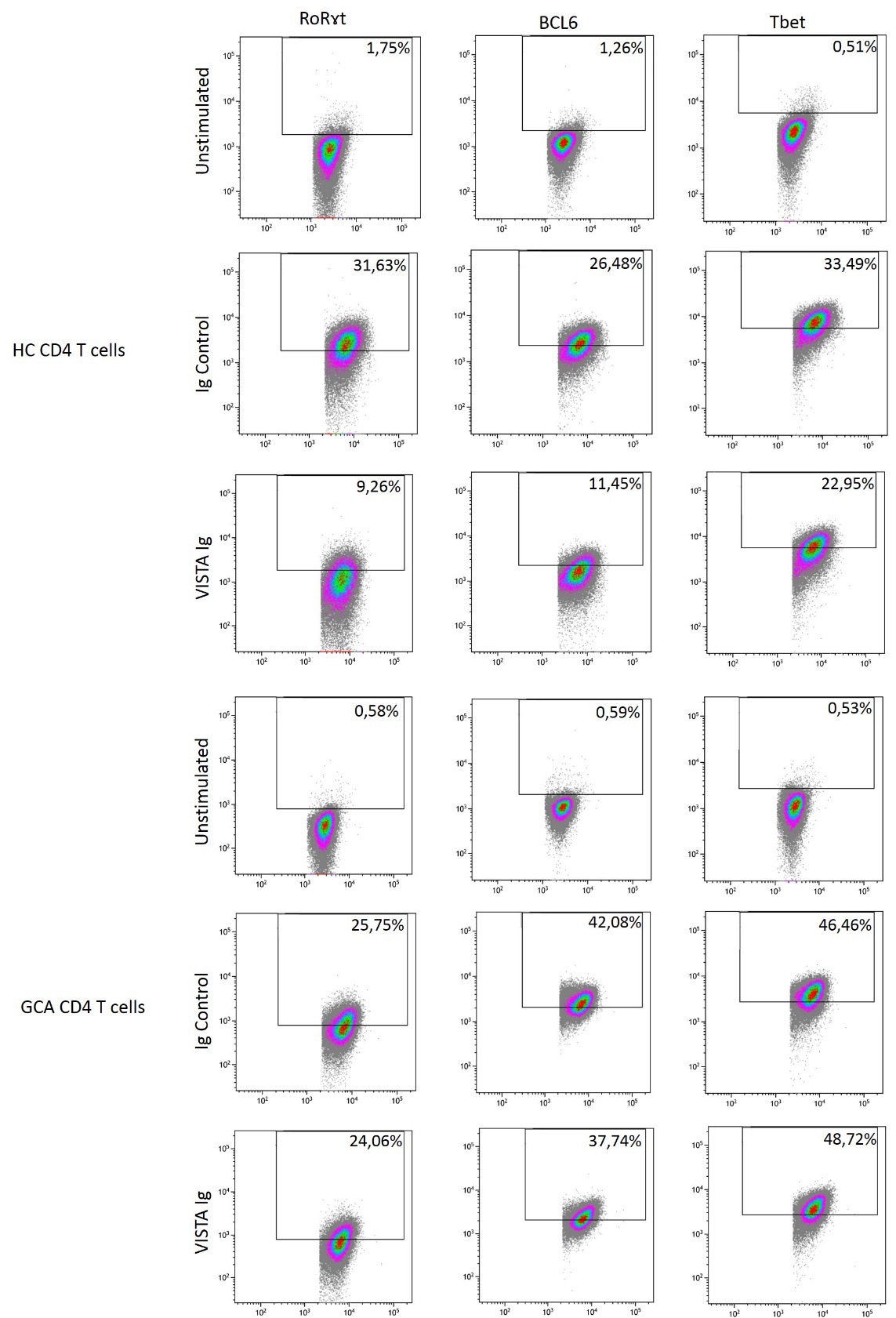
**

**Supplemental Figure S9: Decreased expression of transcription factors by CD4+ T cells following VISTA-Ig engagement of HC but not GCA patients.** Representative histograms showing expression of RoRɤt, BCL6 and Tbet by CD4+ T cells of (A) HC and (B) GCA patients after 5 days of culture with coated anti-CD3 together with control Ig (gray) or VISTA-Ig (red) and soluble anti-CD28.

**
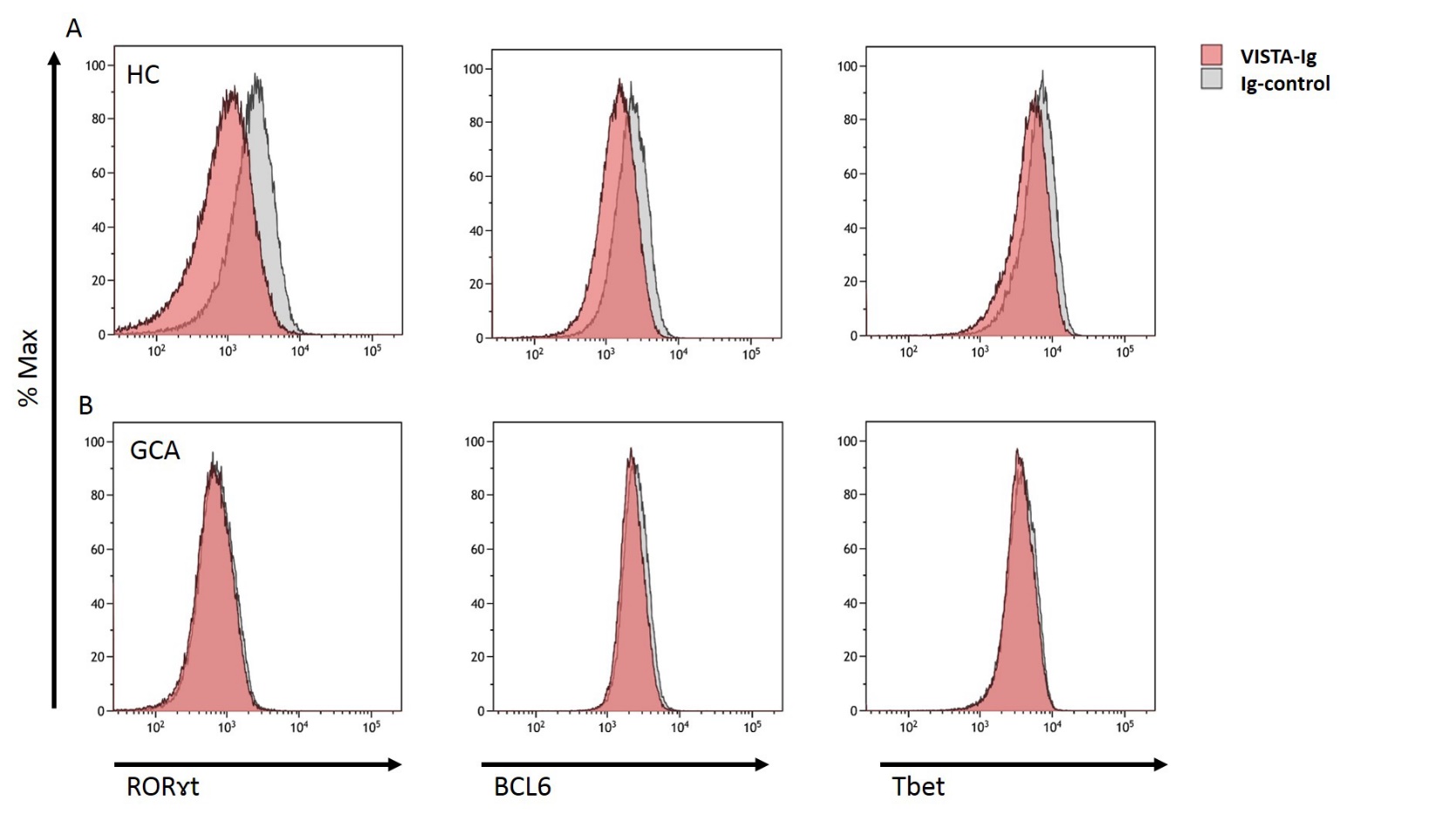
**
